# Reverse phase protein array (RPPA) combined with computational analysis to unravel relevant prognostic factors in non- small cell lung cancer (NSCLC): a pilot study

**DOI:** 10.18632/oncotarget.18480

**Published:** 2017-06-14

**Authors:** Vienna Ludovini, Rita Chiari, Lorenzo Tomassoni, Chiara Antonini, Elisa Baldelli, Sara Baglivo, Annamaria Siggillino, Francesca Romana Tofanetti, Guido Bellezza, K. Alex Hodge, Emanuel Petricoin, Mariaelena Pierobon, Lucio Crinò, Fortunato Bianconi

**Affiliations:** ^1^ Medical Oncology, S. Maria Della Misericordia Hospital, Perugia, Italy; ^2^ Department of Engineering, University of Perugia, Perugia, Italy; ^3^ Center for Applied Proteomics and Molecular Medicine, George Mason University, Manassas, VA, USA; ^4^ Department of Experimental Medicine, Section of Anatomic Pathology and Histology, Perugia, Italy; ^5^ Department of Medical Oncology, Istituto Scientifico Romagnolo per lo Studio e la Cura dei Tumori (IRST) IRCCS, Meldola, Italy; ^6^ Department of Experimental Medicine, University of Perugia, Perugia, Italy

**Keywords:** advanced NSCLC, reverse phase protein array, computational analysis, prognostic factors, cancer system biology

## Abstract

In this work high throughput technology and computational analysis were used to study two stage IV lung adenocarcinoma patients treated with standard chemotherapy with markedly different survival (128 months vs 6 months, respectively) and whose tumor samples exhibit a dissimilar protein activation pattern of the signal transduction. Tumor samples of the two patients were subjected to Reverse Phase Protein Microarray (RPPA) analysis to explore the expression/activation levels of 51 signaling proteins. We selected the most divergent proteins based on the ratio of their RPPA values in the two patients with short (s-OS) and long (l-OS) overall survival (OS) and tested them against a EGFR-IGF1R mathematical model. The model with RPPA data showed that the activation levels of 19 proteins were different in the two patients. The four proteins that most distinguished the two patients were BADS155/136 and c-KITY703/719 having a higher activation level in the patient with short survival and p70S6S371/T389 and b-RAFS445 that had a lower activation level in the s-OS patient. The final model describes the interactions between the MAPK and PI3K-mTOR pathways, including 21 nodes. According to our model mTOR and ERK activation levels were predicted to be lower in the s-OS patient than the l-OS patient, while the AMPK activation level was higher in the s-OS patient. Moreover, KRAS activation was predicted to be higher in the l-OS *KRAS*-mutated patient. In accordance with their different biological properties, the Moment Independent Robustness Indicator in s-OS and l-OS predicted the interaction of MAPK and mTOR and the crosstalk AKT with p90RSK as candidates to be prognostic factors and drug targets.

## INTRODUCTION

The identification of druggable driver mutations and rearrangements (e.g., EGFR mutations, ALK, ROS1 and RET-fusions) routinely detected in approximately 15% of NSCLCs has dramatically changed the therapeutic approach for lung adenocarcinoma in the last ten years. However, most lung adenocarcinomas are still treated with standard chemotherapy often showing an opposite behavior in terms of treatment efficacy and survival. Therefore, a better understanding of the multiple defects in signaling pathways, that are dysregulated in these non-oncogene addicted NSCLC and play a critical role in their neoplastic phenotype, has the potential to improve outcomes. An important goal of the reverse phase protein array (RPPA), a high throughput proteomic technique, is to provide a map of the signaling pathways involved in cancer survival and progression that are dysregulated in tumor cells. These aberrations could identify novel predictors of response or identify novel targets for therapy [1, 2].Since protein profiling can quantify post-translational modifications (e.g., such as the receptor tyrosine kinases phosphorylation status) that are intimately linked with activation of signaling proteins, signaling network analyses are an important addition to other molecular profiling techniques such as gene expression analysis. The RPPA has been successfully used in NSCLC as well as in other cancers to identify signaling pathway abnormalities, pharmacodynamic markers and proteins associated with therapeutic resistance [3-6]. Cancer systems biology studies complex interaction networks to identify biomarkers of response and to understand drug resistance along with the effect of combination therapies. It integrates patient or laboratory-based -omics data into robust predictive models [7]. The data obtained with high throughput platforms can be used to create bioinformatics models that can then be used to analyze biological data of a different nature [8]. In this study, we combined RPPA and computational analysis of the EGFR-IGF1R pathways to study two stage-IV lung adenocarcinoma patients treated with standard chemotherapy with marked differences in survival (128 months vs 6 months from diagnosis) and whose tumor samples exhibit a distinctive signal transduction pattern. We integrated RPPA data with a mathematical model previously generated to describe key signaling pathways in NSCLC using robustness analysis as a quantitative measurement indicating the cell ability to maintain its functions against internal and external perturbations. Here we present results that might be of great value though they are derived from the analysis of two cases with divergent behavior.

## RESULTS

### Case presentation

**Figure 1:**
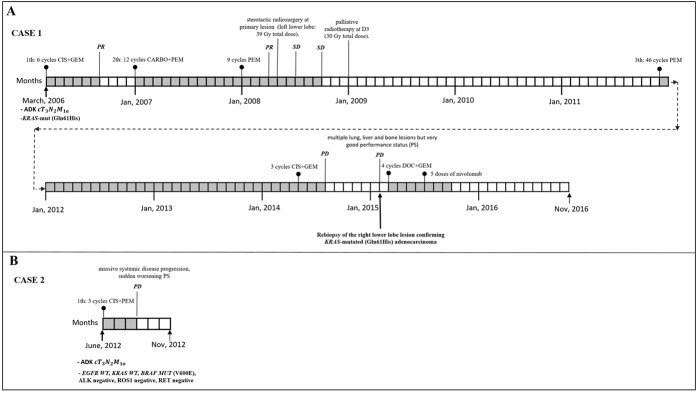
Clinical course, systemic and loco-regional treatment of two cases **A.** Case 1 with stage at diagnosis of lung adenocarcinoma IV [T3N2M1a TNM ver 7.0],*KRAS*-mutated (Q61H). From April to August 2006: six courses of cisplatin plus gemcitabine obtaining partial remission (PR). From January 2007 to September 2009: second line chemotherapy with carboplatin plus pemetrexed for 12 cycles followed by pemetrexed maintenance for 9 cycles obtaining PR after 3 courses followed by radiological stability (SD). In April 2008: stereotactic radiosurgery at primary lesion (left lower lobe: 39 Gy total dose). In January 2009: palliative radiotherapy at D3 (30 Gy total dose). From December 2011 to April 2014: third line chemotherapy with pemetrexed for 46 cycles. From May 2014 to July 2014: 3 courses of carboplatin plus gemcitabine with progression disease (PD). In February 2015: PD with multiple lung, liver and bone lesions but very good performance status (PS). Re-biopsy of the right lower lobe lesion confirming *KRAS*-mutated (Q61H) adenocarcinoma. From March to June 2015: 4 courses of docetaxel plus gemcitabine obtaining a PR. From July to September 2015: 5 doses of nivolumab. From September to November 2016 (date of death): worsening of PS, received best supportive care only. **B.** Clinical course and systemic treatment of case 2: stage at diagnosis of lung adenocarcinoma IV [cT3N2M1a TNM ver 7.0] (video-thorascopic biopsie) with the molecular phenotype being *EGFR WT, KRAS WT, BRAF MUT* (V600E), ALK negative, ROS1 negative, RET negative. From June to August 2012: 3 cycles of first line chemotherapy with cisplatin plus pemetrexed with massive systemic disease progression, sudden worsening PS and death for PD in November 2012.

*Case 1.* A 67-year-old never smoker woman with a *KRAS*-mutated (Gln61His), *EGFR* wild-type (WT), *PI3KCA* WT stage IV [T3N2M1a TNM v.7.0] lung adenocarcinoma, diagnosed in March 2006 and an OS of 128 months (long survival: l-OS) on standard chemotherapy treatment. Her clinical course is described in Figure 1A.
*Case 2.* A never smoker 72-year-old woman diagnosed in June 2012 with stage IV [cT3N2M1a TNM v.7.0] lung adenocarcinoma (video-thoracoscopic biopsies) with the molecular phenotype being *EGFR WT, KRAS WT, BRAF MUT* (V600E), ALK negative, ROS1 negative, RET negative. Her OS was 6 months from diagnosis (short survival; s-OS) with massive systemic disease progression after 3 cycles of first-line chemotherapy with cisplatin plus pemetrexed, sudden worsening of ECOG Performance Status (PS) and death due to disease progression in November 2012 (Figure 1B).

### RPPA and computational analysis

**Figure 2:**
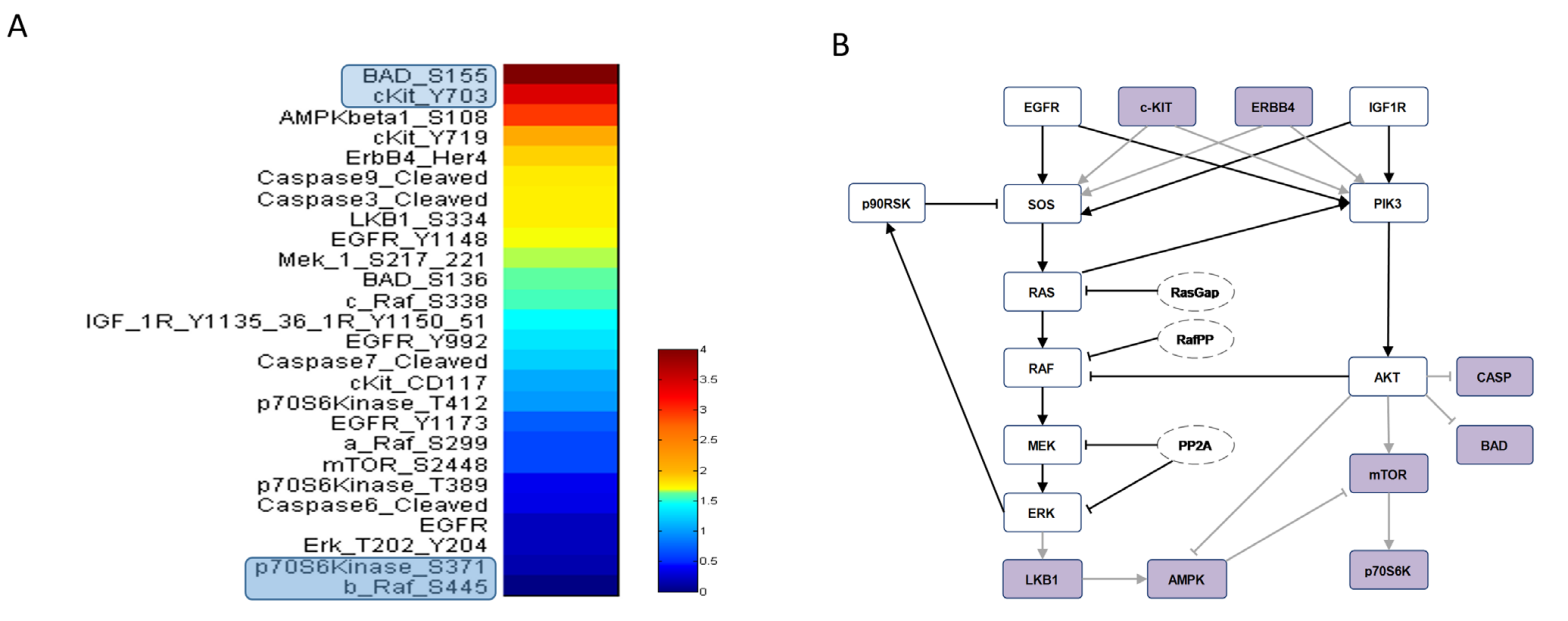
RPPA data and model pathways **A.** RPPA data proteins ratio of s-OS patient vs l-OS patient. **B.** Protein pathways used in the computational analysis: violet nodes are new nodes added to the previous model [9] using RPPA data.

**Figure 3:**
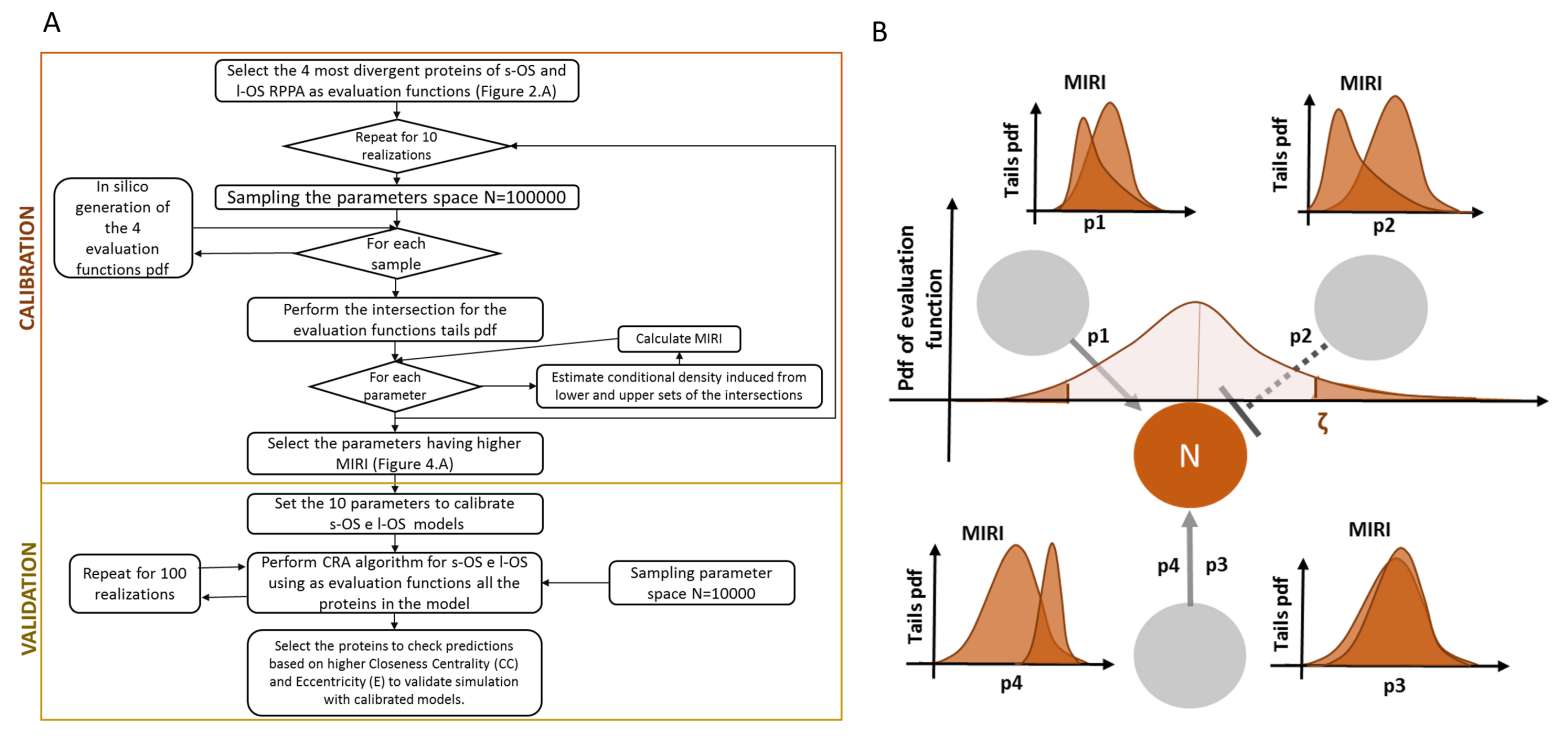
Validation and calibration flow diagram based on MIRI **A.** Flow diagram of calibration and validation algorithm. **B.** An example of the relationship between evaluation function and the Moment Independent Robustness Indicator (MIRI).

**Figure 4:**
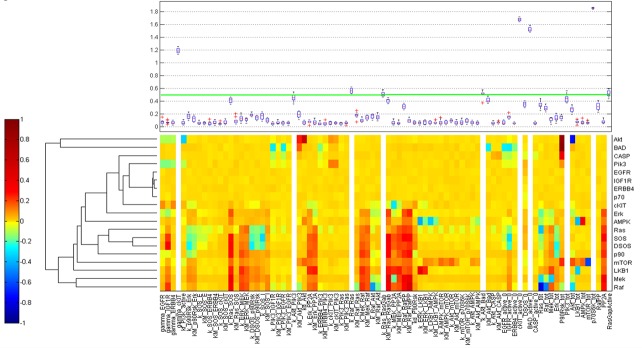
Calibration results Heat map of the difference of s-OS MIRI and l-OS MIRI for all the proteins and parameters after calibration. The box plot shows the selected parameters in the calibration procedure according to their MIRI index obtained using the RAF c-KIT, p70 and Bad RPPA data.

**Figure 5:**
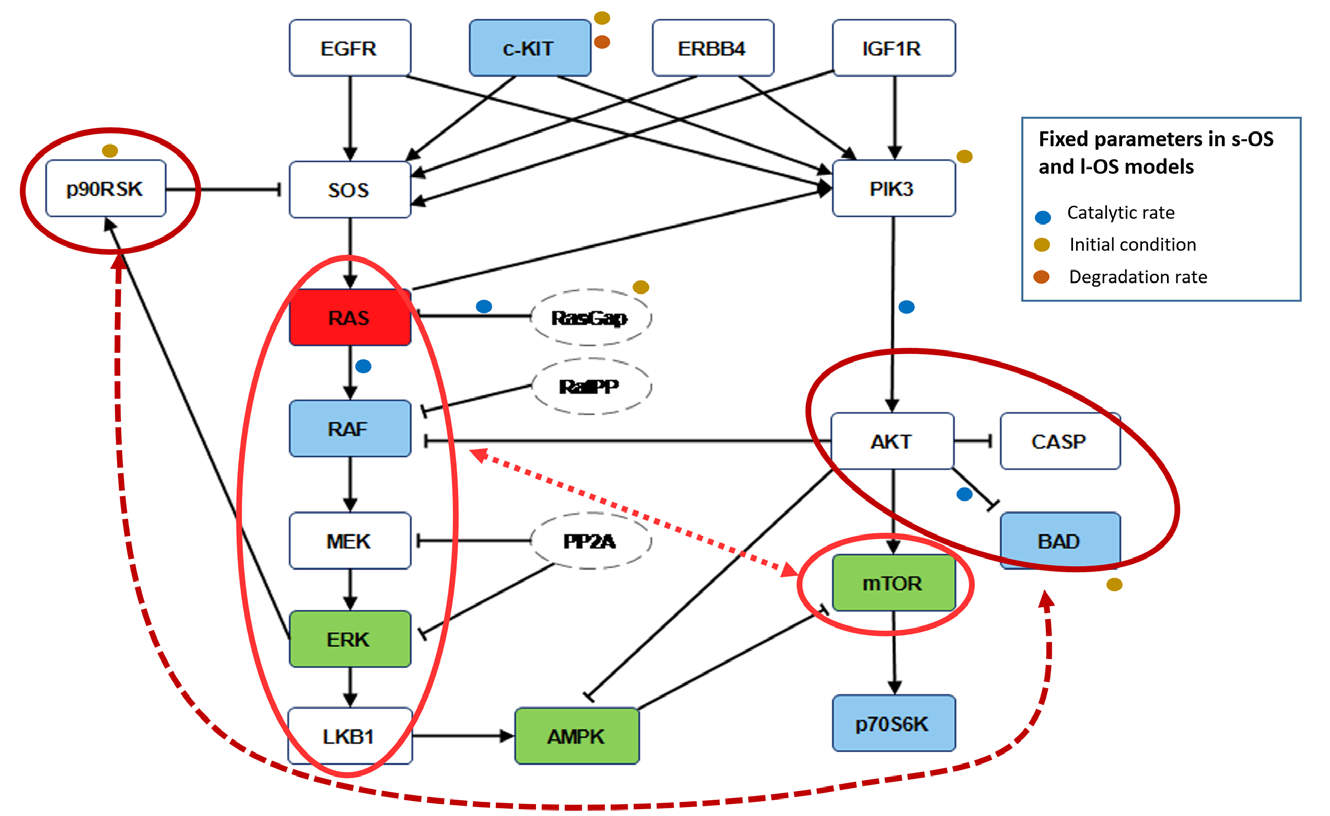
Robustness features Blue nodes in the pathways are the proteins used in the calibration procedure to obtain the s-OS patient and l-OS patient models. Green nodes are selected to validate the model prediction compared with RPPA data. Red node is the KRAS protein not available as RPPA measure but sequenced in both patients. The red circles and the dashed lines in the pathways denote the nodes that influence the higher global robustness in the s-OS patient vs the l-OS patient. The fixed parameters in s-OS and l-OS calibrated models are related to the signal pathways.

**Figure 6:**
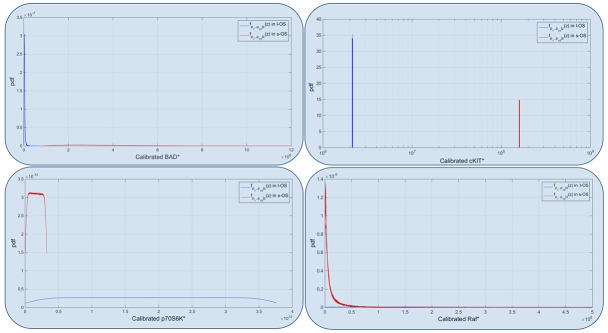
Model calibrated Calibrated probability densities for Bad, c-KIT, p70 and RAF proteins in s-OS and l-OS patients, generated fixing 10 parameters and sampling 76 parameters with hypercube of 10000 samples and repeated for 100 different realizations. Blue boxes are related to Figure 5 and are the proteins used to calibrate the two models.

**Figure 7:**
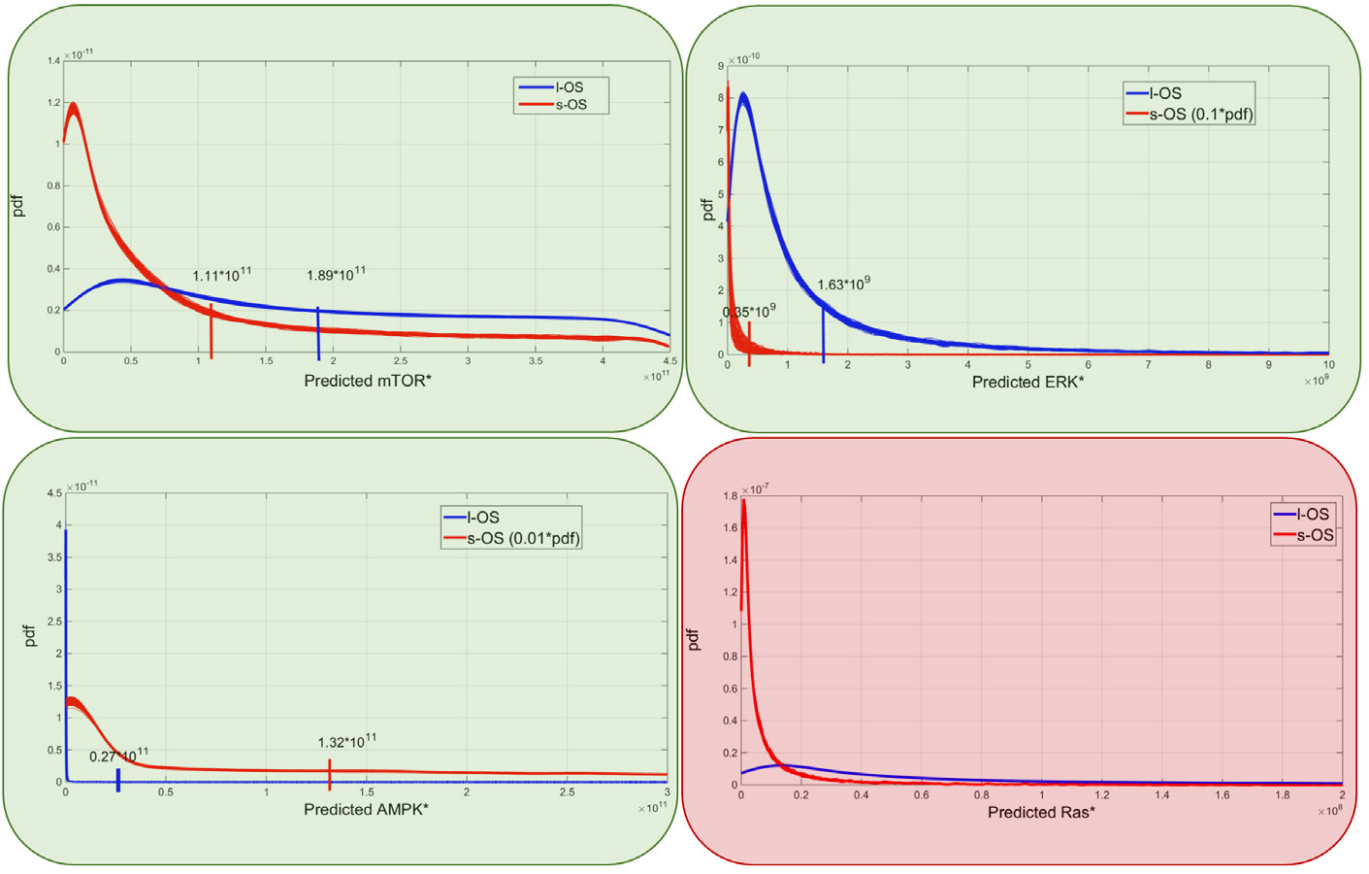
Model predictions Predicted probability densities for ERK, AMPK, mTOR and KRAS proteins in s-OS and l-OS patients, generated fixing 10 parameters and sampling 76 parameters with hypercube of 10000 samples and repeated for 100 different realizations. Green and red boxes are related to Figure 5 and are the proteins selected to validate and produce prediction of the models, respectively.

RPPA result analysis showed that 20 endpoints (active sites and/or total proteins) were differently activated in the two patients. Among these, BAD S155/136 and c-KIT Y703/719 proteins had higher activation levels in the s-OS patient while p70S6 S371/T389 and b-RAF S445 proteins had lower activation levels in the s-OS patient (Table 1 and Figure 2A heat map). The computational model included 21 relevant proteins (Figure 2B). Thirteen proteins were in our previous mathematical model [9] and 8 proteins were added in the pathways using both literature information and Kyoto Encyclopedia of Genes and Genomes (KEGG) Pathway Database (Table 2 and Section 1 in Supplementary Material) [10]. Following the flow diagram in Figure 3A, calibration algorithm, we used BAD, c-KIT, p70S6 and b-RAF to personalize the general model of the patient with s-OS and l-OS, respectively. The calibration procedure based on the Conditional Robustness Algorithm (CRA) [11] and Moment Independent Robustness Indicator (MIRI) (Figure 3B) produced 10 parameters that had MIRI index higher than the cut-off threshold (Box plot in Figure 4, green threshold). In the validation process (Figure 3A), we fixed the 10 parameters to fit the RPPA data using their specific induced probability density function (pdf) tails for s-OS and l-OS patients, respectively (Figure 5 and Table S7 in Supplementary Material). The s-OS patient and the l-OS patient personalized models were simulated *in silico* and MIRIs for all 19 nodes versus all non-fixed parameters were generated. The heat map in Figure 4 shows the difference between the MIRI of the s-OS patient and l-OS patient. The higher MIRI indicates higher robustness of the network proteins. The overall robustness of the s-OS patient was also higher than the l-OS patient. The dendrogram in Figure 4 clustered the proteins that mainly contribute to the higher robustness of the s-OS patient. These proteins were represented with two main interactions between proteins included in the mTOR and MAPK cascades, namely AKT and p90RSK pathways (Figure 5). We used the endpoints ERK, AMPK and mTOR, having higher closeness centrality (CC) and eccentricity (E) in the pathway to validate the personalized models (Green nodes in Figure 5 and Section 3 in Supplementary Material). We generated *in silico* measures of the proteins BAD, c-KIT, p70S6 and b-RAF and of the proteins ERK, AMPK and mTOR used for the calibration and validation, respectively. Figures 6 and 7 show the conditional pdfs for the *in silico* measures of the evaluation functions that are the area under the curve of the chosen proteins. These evaluation functions are a computational index of the protein activation levels. The model generated the expected distributions for the proteins BAD, c-KIT, p70S6 and b-RAF (Figure 6). Furthermore, the model predicted their behavior according to RPPA data: mTOR and ERK protein levels were lower in the s-OS than in the l-OS patient, while AMPK protein level was higher in the s-OS than in the l-OS patient as seen in the original data (Figure 7 green boxes). Moreover, the model predicted higher expression levels of KRAS in the l-OS patient who was found to have a *KRAS* somatic mutation in her tumor sample (Figure 7 red box).

**Table 1:** Antibodies measured in the two cases.

Catalog Number	Antibody	Company	Dilution	l-OS (a.u.)	s-OS (a.u.)	s-OS/l-OS
9297	BAD (S155)*	Cell Signaling	1:100	0.00	15994.50	8.55**
3073	c-Kit (Y703)*	Cell Signaling	1:50	9339.43	33189.87	3.55
4181	AMPKBbeta1 (S108)*	Cell Signaling	1:50	7072.72	20090.45	2.84
3391	cKit (Y719)*	Cell Signaling	1:100	13042.91	26635.48	2.04
4795	ErbB4/HER4 (111B2)*	Cell Signaling	1:50	18196.78	34200.64	1.88
9501	Caspase-9 cleaved (D330)*	Cell Signaling	1:50	16848.22	30031.45	1.78
9661	Caspase-3. cleaved (D175)*	Cell Signaling	1:50	8769.20	15599.59	1.78
3051	LKB1 (S428) *	Cell Signaling	1:100	22925.38	40538.19	1.77
44-792	EGFR (Y1148)*	Invitrogen	1:100	15214.44	25848.29	1.70
9121	MEK1/2 (S217/221)*	Cell Signaling	1:500	43914.49	67507.90	1.54
9295	BAD (S136)*	Cell Signaling	1:50	17890.05	24343.02	1.36
2101	Src family (Y416)	Cell Signaling	1:200	31571.17	42192.58	1.34
9427	c-Raf (S338)*	Cell Signaling	1:200	28001.12	36680.49	1.31
44-1100	PRAS40 (T246)	BioSource	1:1000	45706.67	59874.14	1.31
9455	4E-BP1 (T70)	Cell Signaling	1:200	58104.61	70262.95	1.21
3024	IGF-1 Rec (Y1135/36)/Insulin Rec (Y1150/51)*	Cell Signaling	1:500	20702.29	23860.98	1.15
9181	Elk-1(S383)	Cell Signaling	1:100	20743.74	23388.52	1.13
2235	EGFR (Y992)*	Cell Signaling	1:50	38177.45	41772.79	1.09
9491	Caspase-7. cleaved (D198)*	Cell Signaling	1:50	32532.68	33860.36	1.04
34-8800	c-Kit (CD117)*	Zymed	1:500	31888.47	30333.25	0.95
2808	Survivin (71G4)	Cell Signaling	1:100	21720.25	20373.70	0.94
07-018	p70S6 Kinase (T412)*	Upstate	1:500	12848.73	11684.29	0.91
3597	eIF2alpha (S51)	Cell Signaling	1:500	4514.28	3873.83	0.86
2926	Cyclin D1 (G124-326)	Cell Signaling	1:100	18958.36	15677.78	0.83
2386	IRS-1 (S612)	Cell Signaling	1:200	25591.09	21036.21	0.82
2827	Bcl-2 (S70)	Cell Signaling	1:50	38948.66	30638.11	0.79
9211	p38 MAP kinase (T180/Y182)*	Cell Signaling	1:100	35954.15	28001.12	0.78
44-794	EGFR (Y1173)*	Invitrogen	1:100	18901.57	14472.42	0.77
2632	Histone Deacetylase 3 (HDAC3)	Cell Signaling	1:1000	8358.21	6173.37	0.74
9251	SAPK/JNK (T183/Y185)	Cell Signaling	1:100	66171.19	47098.68	0.71
4431	a-Raf (S299)*	Cell Signaling	1:100	23623.56	16613.99	0.70
2971	mTOR (S2448)*	Cell Signaling	1:100	25336.48	17729.76	0.70
9331	GSK-3alpha/beta (S21/9)	Cell Signaling	1:1000	56954.03	39735.50	0.70
3736	TNF-R1 (C25C1)	Cell Signaling	1:50	9246.50	5302.85	0.57
9451	4E-BP1 (S65)	Cell Signaling	1:50	27173.57	14559.52	0.54
9205	p70S6 Kinase (T389)*	Cell Signaling	1:100	42192.58	20952.22	0.50
4656	Cyclin A (BF683)	Cell Signaling	1:50	33189.87	16171.41	0.49
9761	Caspase-6 cleaved (D162)	Cell Signaling	1:50	23155.79	10938.02	0.47
2772	Bax	Cell Signaling	1:200	14016.64	6130.30	0.44
9134	Stat3 (S727)	Cell Signaling	1:100	64215.49	25591.09	0.40
2232	EGFR*	Cell Signaling	1:100	60475.90	23155.79	0.38
9101	ERK 1/2 (T202/Y204)*	Cell Signaling	1:1000	35242.23	13160.82	0.37
9208	p70S6 Kinase (S371) *	Cell Signaling	1:50	8324.85	2855.49	0.34
Sc-23950	Tubulin. alpha acetylated (6-11B-1)	SantaCruz	1:200	32208.97	9595.02	0.30
2696	b-Raf (S445)*	Cell Signaling	1:50	25336.48	6075.38	0.24
2772	Bak	Cell Signaling	1:200	4289.82	850.65	0.20
Sc-32793	NQO1	SantaCruz	1:200	12456.53	2109.06	0.17
610203	Cox-2	BD	1:200	41357.13	6136.44	0.15
SPA-894	Heme Oxygenase 1	Stressgen	1:500	45399.93	5084.75	0.11
3104	Smad2 (S245/250/255)	Cell Signaling	1:100	33523.44	3203.50	0.10
2105	Src (Y527)	Cell Signaling	1:200	36680.49	0.00	0.00

The records with * are the antibodies used in the model that are related to the EGFR-IGF1R model [9] according to KEGG Pathway Database.

*are the antibodies included in the mathematical model

**ratio is in log10 scale

**Table 2:** List of the proteins from model [9], from KEGG and protein excluded from the model.

Proteins from model [9]	Proteins from KEGG	Proteins excluded from the model
EGFR	ERBB4	SMAD2
IGF1R	cKIT	COX2
SOS	CASP	NQO1
p90RSk	BAD	BAK
RAS	LKB1	Tubulin-alpha-acetylated
RAF	AMPK	STAT3
MEK	p70S6k	Heme-Oxygenase1
ERK		
RasGAP		
RafPP		
PP2A		
PIK3		
Akt		

## DISCUSSION

This work combined high throughput proteomic analysis, namely RPPA, and computational analysis to study the complex signaling network of two stage IV (cT3N2M1a) lung adenocarcinoma patients treated with standard chemotherapy with an opposite clinical behavior in term of OS. The molecular profiling of their tumors showed that the l-OS patient was *KRAS*-mutated (Q61H) and the s-OS patient was *BRAF-*mutated (V600E), but neither of them had access to targeted therapy. The RPPA was used to calibrate the model in both patients and to validate predictions of the model. The main predicted information was the higher overall robustness for the s-OS patient in all the proteins included in the model. This could explain the shorter survival of the s-OS patient whose tumor was more aggressive and the signaling network had a structurally higher probability to maintain the neoplastic phenotype and overcome the effects of standard chemotherapy.

The MIRI applied to each protein in the calibrated models were a measure of robustness of the model. The proteins in the pathway with higher MIRI were the ones that contributed to the overall robustness of the tumor. The s-OS had higher MIRIs than l-OS and this can be associated with a more adaptable resistance of the cancer cells to the therapeutic treatment. Ideally, in the s-OS patient only a multi-target treatment could have helped to obtain a better response. The higher robustness was within two main interactions: the MAPK cascade including KRAS, b-RAF, MEK, ERK and LKB1 that interacts with mTOR and AKT, BAD and Caspase proteins that crosstalk with p90RSK [12-14]. While the mTOR and MAPK cascade interactions are known to play a relevant role in lung cancer cell signal transductions [15], to our knowledge the interaction between p90RSK and BAD in the anti-apoptotic process has never been described and might represent a potential prognostic factor in lung cancer.

The computational model helps to interpret RPPA data even when single events are measured.

In the validation process, the s-OS and l-OS pdf of the active proteins were used. The relative values of RPPA data in s-OS and l-OS were comparable with the relative pdf of s-OS and l-OS generated i*n silico*. If the RPPA value of a protein was lower in the s-OS rather than in the l-OS, we expected the pdf-mean value of the s-OS evaluation function to be lower compared to that of l-OS.

The calibrated models for s-OS and l-OS had 10 parameters fixed in opposite conditions of the induced tails distributions to fit the 4 divergent measured proteins. The other 76 parameters were free to move in the validation process using 10000 samples for each of 100 realizations. Fifteen percent of the active proteins in the pathway influenced the tumor growth of the two patients in opposite ways. The simulations of the calibrated models were stable as can inferred from the small variations of the distributions among the 100 realizations. Our calibration and validation algorithms were based on only two patient’s RPPA data and this could be a critical point of the study. It could be solved by collecting data of similar patients and performing a statistical analysis to select the most divergent proteins.

The model prediction of KRAS level being higher in the l-OS patient, together with the somatic mutation and with high ERK activation, could lead to the activation of the senescence program of the cancer cells as has been validated in human colon adenocarcinoma cancer models [16]. The mTOR and ERK protein expression that in RPPA data were lower in the s-OS patient than in the l-OS one was confirmed with simulations of the calibrated models.

The nonlinear interactions in the signaling network produced complex behaviors and the computational approach is crucial to discovering this complexity and transferring results to clinical application. The proposed method could be used to stratify patients with similar clinical pathological tumor classification according to the active protein profiles at diagnosis. Using the most divergent protein expression among the clusters obtained from RPPA data, we can calibrate the network and study the pathways associated with the clusters. The use of the CRA algorithm in the calibrated networks will predict the proteins playing the main roles in cancer robustness and these predictions could be used to increase patient survival with personalized drug treatments. Our approach could be applied to tumors that are genetically similar but have different protein profiles. This could be done by using RPPA technology that is a high sensitivity system and also by using the computational method that has an internal control of the distribution stability among independent realizations generated *in silico*. However, when the protein expressions of the samples are very similar, our methodology is not applicable.

In conclusion, coherently with their different biological properties, the Moment Independent Robustness Indicator in s-OS vs l-OS patients confirmed the MAPK cascade interaction with mTOR and predicted new crosstalk of AKT, BAD and Caspase proteins with p90RSK that could be a candidate for prognostic factors or drug targets.

## MATERIALS AND METHODS

### Tumor and data collection

Two patients followed at the Medical Oncology Department of the S. Maria della Misericordia Hospital with a diagnosis of stage IV lung adenocarcinoma having a short (s-OS: 6 months) and long (l-OS: 128 months) overall survival, respectively, were identified from the institutional database. Pathologic data, tumor genotype, treatment type, and radiological parameters were gathered from retrospective chart extraction. The pilot study was approved by the local Ethics Committee and was conducted in accordance with ethical principles of the latest version of the Declaration of Helsinki. Written informed consent for RPPA analysis was obtained from the two patients included in the pilot study.

### Reverse phase protein microarray

Formalin-fixed, paraffin-embedded (FFPE) tumor samples were subjected to Laser Capture Microdissection and RPPA analysis to explore the expression/activation levels of 51 signaling proteins using a previously described protocol [17]. Proteins and phosphoproteins were selected based on their involvement in key signaling pathways or cellular functions, including the: (1) PI3K/AKT/mTOR pathway, (2) MAPK pathway, (3) EGFR pathway, (4) LKB1 pathway, (5) RAF pathway.

### Computational analysis based on RPPA data

#### Mathematical model

We developed an ordinary differential equations (ODEs) model starting from the most relevant proteins involved in the signaling pathways studied. Michaelis-Menten kinetics, mass action law and conservation law were combined to write the equations (see Section 1 in Supplementary Material). We selected the most divergent proteins based on the ratio of RPPA values in the two patients (s-OS vs l-OS) and used them to build the ODEs model (Table 1, Figure 2A). We included the proteins inserted in our pre-existing EGFR-IGF1R mathematical model [9] and the pathways were extended using KEGG PATHWAY Database combined with a literature revision in lung cancer pathways (Figure 2B). The isolated proteins were excluded because they did not contribute to the model dynamics (Table 2 and Section 1 and 2 in Supplementary Material) [10].

#### Model calibration based on conditional robustness

The CRA proposed in [11] was an iterative procedure to study the dynamical model robustness perturbing the model parameters. CRA is based on the probability distribution of the evaluation function and MIRI that measures the dissimilarity of pdf tails. The evaluation function tails induce for each parameter of the model the pdf tails and their dissimilarity is measured through the MIRI. As an example, Figure 3B shows the induced pdf tails and their MIRI for a node N (proteins) that interact with 3 other nodes through the 3 edges described in the ODEs with 4 parameters: p1, p2, p3 and p4.

The calibration and validation processes are presented in the flow chart in Figure 3B. We selected the 4 most divergent proteins of s-OS and l-OS RPPA data as evaluation functions (Figure 2A) and we performed *in silico* simulations from the hypercube of parameters space generating the pdf of the selected proteins. We evaluated the MIRI for all parameters using the intersection of the evaluation function tails. The calibration process was repeated for 10 realizations using 100000 samples for each of them (Box plot Figure 4).

After calibration, we fixed 10 parameters with opposite conditions for s-OS and l-OS simulations having higher MIRI (Green line in the box plot Figure 4). We performed a CRA algorithm using as evaluation functions all the proteins in the model and we clustered the difference of s-OS and l-OS MIRIs to explore the most relevant proteins in network robustness (heat map with row dendrograms in Figure 4 and Figure 5). We selected the network proteins with higher closeness centrality (CC) and eccentricity (E) to compare the simulations with RPPA data (Green nodes in Figure 5). We checked the calibration results comparing the conditional distributions in s-OS and l-OS (Figure 6) and we validated the model predictions comparing the simulation results and RPPA data (Figure 7 and Section 3 in Supplementary Material).

The mathematical model and the computational analysis were performed using Python programming language (Supplementary Data).

## SUPPLEMENTARY MATERIALS TABLES







## Abbreviations

RPPA: Reverse Phase Protein Microarray
NSCLC: non-small cell lung cancer
MIRI: Moment Independent Robustness Indicator
s-OS: short overall survival
l-OS: long overall survival
CRA: Conditional Robustness Algorithm
ECOG: Eastern Cooperative Oncology Group
PS: Performance Status
PDF: probability density function
KEGG: Kyoto Encyclopedia of Genes and Genomes
CC: closeness centrality
E: eccentricity
ODEs: ordinary differential equations
