# Supplementary material for: Reverse phase protein array (RPPA) combined with computational analysis to unravel relevant prognostic factors in non- small cell lung cancer (NSCLC): a pilot study
Source: Oncotarget. 2017 Jun 14;8(47):83343–53. doi: 10.18632/oncotarget.18480 (PMC5669974; doi:10.18632/oncotarget.18480)
Supplement: Supplementary file 1 [file oncotarget-08-83343-s001.pdf]

Reverse phase protein array (RPPA) combined with computational analysis to unravel relevant prognostic factors in non- small cell lung cancer (NSCLC): a pilot study

SUPPLEMENTARY MATERIALS

1 Model Extension

The EGFR-IGF1R network defined in [1,2] is a key pathway in cancer pathogenesis and progression, mainly in Non Small Cell Lung Cancer (NSCLC). It describes interactions between epidermal growth factor receptor (EGFR) and insulin-like growth factor 1 receptor (IGF1R), together with their downstreams Mitogen-activated protein kinases (MAPK) and the inositol phospholipid kinases axis (PIK3) (Fig S1). The corresponding Ordinary Differential Equations (ODEs) model has 10 state variables, 3 input signals and a parameter vector of 39 entries. First of all, we extended this model adding 8 further proteins from the RPPA dataset presented in the main text. The dataset comprises measures of 51 signaling proteins on two tumor samples of stage IV lung adenocarcinoma. In order to identify the most discriminant proteins between the two samples, we calculated the ratio of each protein value among the two patients (s-OS vs l-OS). This strategy was necessary because RPPA values cannot be compared among different proteins but only among different samples of the same protein. Indeed, these values are non dimensional since they do not quantify a specific physical quantity but they are an index of the expression level of a protein. This index is strictly related with the antibody-analyte affinity constant. From this classification, we selected the first 10 endpoints with high ratio and the last 10 with low ratio, as shown in Table S2 and S3. Then, from this set of 20 proteins, we excluded those that were already in the model and, for the remaining ones, we executed a bibliographic research in order to find out only those related with the EGFR-IGF1R model in [1,2]. From [3] and [4] we extended the previous model adding 5 nodes and 7 interactions:

- ERK → LKB1
- LKB1 → AMPK
- mTOR → p70S6K
- AKT → mTOR
- AMPK ⊣ mTOR
- AKT ⊣ BAD
- AKT ⊣ CASP.

In order to validate the insights presented above, we also queried the KEGG PATHWAY Database, a reference knowledge base containing interaction maps of signal transduc-tion, cellular processes and human diseases [5]. In this database, we entered, as key-words, the names of the 15 selected endpoints one after the other and chose ’hsa’ (Homo Sapiens) as organism. From the resulted pathways, we analyzed only those relevant for cancer disease. Through view inspection of the pathways reported in Table S1, we not only confirmed some of the reactions inserted from the literature but we also included 3 further nodes and 5 further interactions:

- cKIT → SOS
- cKI → PIK3

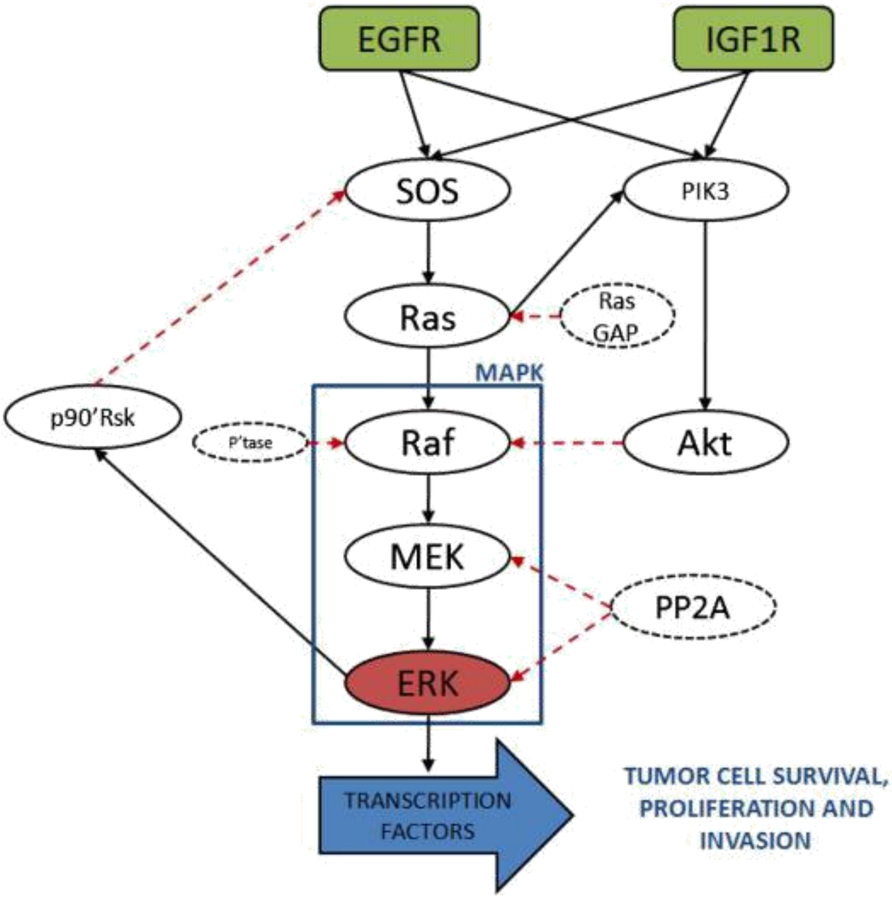

Figure S1: EGFR-IGF1R pathways [1] [2].

- ERBB4 ⟶ SOS
- ERBB4 ⟶ PIK3
- AKT ⊣ AMPK.
-

Table S4 summarizes the procedure explained above.

2 Model Description

The resulting pathway (shown in the main text) has been modeled using ODEs, in order to investigate the time behavior of all the species included in it. Two widely accepted kinetic laws were used to model biochemical reactions: the law of mass action and Michaelis-Menten kinetics [6]. The first one is used to describe the degradation of a species while the second one models the activation or inhibition of a protein through an enzyme. As an example of the mass action law, we report the case of EGFR degradation, according to the following chemical reaction:

$$EGFR_{active} \xrightarrow{\gamma_{EGFR}} EGFR$$

The correspoding differential equation is:

$$\frac{dEGFR_{active}}{dt} = -\gamma_{EGFR} * EGFR_{active}$$

where γ_{EGFR} is the degradation rate of the receptor. In order to show the application of Michaelis Menten kinetics, let consider the case of ERK activation by MEK described as:

$$MEK_{active} + ERK \xrightarrow{k_{MEK:ERK},\ KM_{MEK:ERK}} ERK_{active} + MEK_{active}$$

This reaction is mathematically translated into the following differential equation:

$$\frac{dERK_{active}}{dt} = \frac{k_{MEK:ERK} * MEK_{active} * ERK^{T} - ERK_{active}}{KM_{MEK:ERK} + (ERK^{T} - ERK_{active})}$$

where $k_{MEK:ERK}$ is the catalytic rate, $KM_{MEK:ERK}$ is the Michaelis Menten constant and ERK^T is the total concentration of protein ERK. Notice that the above Michaelis Menten formulation explicits the possible time-varying enzyme concentration [7]. Since our RPPA dataset contains mainly measures of phosphorylated proteins, the state variables included in the model describe only their active form. As a consequence, we wrote the model considering the conservation law and assuming (as it is common in this field) that the total amount of each protein is constant and equal to x_i^T i=7,...17. Therefore regarding the above example the following constraint holds:

$$[ERK] + [ERK_{active}] = [ERK^{T}]$$

We also assumed that the total mean values of the number of molecules in the cells do not change significantly in NSCLC with respect to normal cells. The complete system has 19 state variables, 3 input signals and 65 kinetic parameters. The corresponding ODEs are reported below.

$$\dot{x_1} = -p_1 x_1$$

$$\dot{x_2} = -p_2 x_2$$

$$\dot{x_3} = -p_3 x_3$$

$$\dot{x_4} = -p_4 x_3$$

$$\dot{x_5} = p_{10} x_3 \frac{x_6}{p_{11} + x_6} + p_{12} x_4 \frac{x_6}{p_{13} + x_6} + p_8 x_1 \frac{x_6}{p_9 + x_6} + p_{20} x_2 \frac{x_6}{p_{21} + x_6} - p_{18} x_{11} \frac{x_5}{p_{19} + x_5}$$

$$\dot{x_6} = -p_{10} x_3 \frac{x_6}{p_{11} + x_6} - p_{12} x_4 \frac{x_6}{p_{13} + x_6} - p_8 x_1 \frac{x_6}{p_9 + x_6} - p_{20} x_2 \frac{x_6}{p_{21} + x_6} + p_{18} x_{11} \frac{x_5}{p_{19} + x_5}$$

$$\dot{x_7} = p_{14} x_5 \frac{(x_7{}^T - x_7)}{p_{15} + (x_7{}^T - x_7)} - p_{43} u_3 \frac{x_7}{p_{44} + x_7}$$

$$\dot{x_8} = p_{37} x_7 \frac{x_8{}^T - x_8}{p_{38} + (x_8{}^T - x_8)} - p_{47} u_1 \frac{x_8}{p_{48} + x_8} - p_{41} x_{13} \frac{x_8}{p_{42} + x_8}$$

$$\dot{x_9} = p_{39} x_8 \frac{x_9{}^T - x_9}{p_{40} + (x_9{}^T - x_9)} - p_{45} u_2 \frac{x_9}{p_{46} + x_9}$$

$$\dot{x_{10}} = p_{16} x_9 \frac{x_{10}{}^T - x_{10}}{p_{17} + (x_{10}{}^T - x_{10})} - p_{29} u_2 \frac{x_{10}}{p_{30} + x_{10}}$$

$$\dot{x_{11}} = p_6 x_{10} \frac{x_{11}{}^T - x_{11}}{p_7 + (x_{11}{}^T - x_{11})} - p_{49} x_{11}$$

$$\dot{x_{12}} = p_{22} x_2 \frac{x_{12}{}^T - x_{12}}{p_{23} + (x_{12}{}^T - x_{12})} + p_{24} x_1 \frac{x_{12}{}^T - x_{12}}{p_{25} + (x_{12}{}^T - x_{12})} + p_{31} x_3 \frac{x_{12}{}^T - x_{12}}{p_{32} + (x_{12}{}^T - x_{12})} + p_{33} x_4 \frac{x_{12}{}^T - x_{12}}{p_{34} + (x_{12}{}^T - x_{12})} +$$

$$+ p_{35} x_7 \frac{x_{12}{}^T - x_{12}}{p_{36} + (x_{12}{}^T - x_{12})} - p_5 x_{12}$$

$$\dot{x_{13}} = p_{26} x_{12} \frac{x_{13}{}^T - x_{13}}{p_{27} + (x_{13}{}^T - x_{13})} - p_{28} x_{13}$$

$$\dot{x_{14}} = p_{50} x_{10} \frac{x_{14}{}^T - x_{14}}{p_{51} + (x_{14}{}^T - x_{14})}$$

$$\dot{x_{15}} = p_{52} (x_{14}{}^T - x_{14}) \frac{x_{15}{}^T - x_{15}}{p_{53} + (x_{15}{}^T - x_{15})} - p_{60} x_{13} \frac{x_{15}}{p_{61} + x_{15}}$$

$$\dot{x_{16}} = p_{56} x_{13} \frac{x_{16}{}^T - x_{16}}{p_{57} + (x_{16}{}^T - x_{16})} - p_{54} x_{15} \frac{x_{16}}{p_{55} + x_{16}}$$

$$\dot{x_{17}} = p_{58} x_{16} \frac{x_{17}{}^T - x_{17}}{p_{59} + (x_{17}{}^T - x_{17})}$$

$$\dot{x_{18}} = -p_{62} x_{13} \frac{x_{18}}{p_{63} + x_{18}}$$

$$\dot{x_{19}} = -p_{64} x_{13} \frac{x_{19}}{p_{65} + x_{19}}$$

Table S5 and Table S6 list names and nominal values of initial and total concen-trations of variables, input signals and kinetic parameters. We did not change nominal values of parameters in [1,2]. New parameters introduced with the extension proce-dure were set according to the following rules of thumb. Degradation constants and initial concentrations of the receptors cKIT and ERBB4 are equal to those of EGFR and IGF1R. To the kinetic constants of the added activation/inhibition reactions, we assigned values around the center of the interval between the minimum and the maxi-mum order of

magnitude of the kinetic parameters in [1,2]. We used the same logic for Michaelis-Menten constants and total concentrations.

3 Model Validation

Through the calibration procedure explained in the main text, we selected 10 parame-ters which were used to calibrate the mathematical model for each of the two patients. Table S7 shows parameter values returned by the algorithm, for the l-OS and s-OS patients. In order to validate our model, we tested its predictions on a validation set, i.e. a set of proteins included both in the model and in the RPPA dataset but not used for the calibration. These proteins are: EGFR, IGF1R, ERBB4, CASP, MEK, ERK, LKB1, AMPK and mTOR. From this set we first excluded EGFR, IGF1R and ERBB4 because, since they are receptors, they are at the top of the signaling cascade and their temporal behavior is not affected by the 10 fixed parameters. Each of their equations contains only the corresponding degradation parameter g which has the same value in both patients. Second, we kept out CASP protein, because, in the RPPA dataset, differ-ent members of the Caspase family (CASP3, CASP6 and CASP9) exhibit incoherent activation levels. Finally, among the remaining ones we selected the 3 most relevant proteins using a combination of two well-known centrality metrics: the closeness cen-trality (CC) and the eccentricity (E). Consider a graph G defined as G=(N,E) where N is the number of vertexes and E the number of edges, for a given node n 2 G, the close-ness centrality index is the reciprocal of the sum of all shortest paths between the node n and all other nodes, while the eccentricity is the reciprocal of the longest shortest path between node n and all other nodes. We calculated these measures with CentiScaPe [8], a Cytoscape [9] plug-in that computes several network centrality parameters for identifying network nodes, relevant from a topological point of view. Table S8 shows the resulting values of CC and E calculated by the software for ERK, AMPK, mTOR, LKB1 and MEK. Thus, we validated our model on the first 3 proteins of this rank, ERK, AMPK and mTOR (Table S9).

References

1. Bianconi F, Baldelli E, Ludovini V, Crinò L, Flacco A, Valigi P. Computational model of EGFR and IGF1R pathways in lung cancer: a Systems Biology approach for Translational Oncology. Biotechnol Adv. 2012; 30: 142–53.

2. Bianconi F, Chelliah V. BIOMD0000000427 - Bianconi2012 - EGFR and IGF1R pathway in lung cancer. 2013. Available from http://www.ebi.ac.uk/biomodels-main/BIOMD0000000427.

3. Karachaliou N, Mayo C, Costa C, Magrí I, Gimenez-Capitan A, Molina-Vila MA, Rosell R. KRAS mutations in lung cancer. Clin Lung Cancer. 2013; 14: 205–14.

4. Martini M, De Santis MC, Braccini L, Gulluni F, Hirsch E. PI3K/AKT signaling pathway and cancer: an updated review. Ann Med. 2014; 46: 372–83.
5. Kanehisa M, Araki M, Goto S, Hattori M, Hirakawa M, Itoh M, Katayama T, Kawashima S, Okuda S, Tokimatsu T, Yamanishi Y. KEGG for linking genomes to life and the environment. Nucleic Acids Res. 2007; 36: D480–4.
6. Lillacci G, Khammash M. Parameter estimation and model selection in computational biology. PLOS Comput Biol. 2010; 6:e1000696.
7. Orton RJ, Adriaens ME, Gormand A, Sturm OE, Kolch W, Gilbert DR. Computational modelling of cancerous mutations in the EGFR/ERK signalling pathway. BMC Syst Biol. 2009; 3: 100.
8. Scardoni G, Petterlini M, Laudanna C. Analyzing biological network parameters with CentiScaPe. Bioinformatic. 2009; 25: 2857–9.
9. Shannon P, Markiel A, Ozier O, Baliga NS, Wang JT, Ramage D, Amin N, Schwikowski B, Ideker T. Cytoscape : A Software Environment for Integrated Models of Biomolecular Interaction Networks Cytoscape : A Software Environment for Integrated Models of Biomolecular Interaction Networks. Genome Res. 2003; 13:2498-504 .
