# Supplementary material for: Reverse phase protein array (RPPA) combined with computational analysis to unravel relevant prognostic factors in non- small cell lung cancer (NSCLC): a pilot study
Source: Oncotarget. 2017 Jun 14;8(47):83343–53. doi: 10.18632/oncotarget.18480 (PMC5669974; doi:10.18632/oncotarget.18480)
Supplement: Supplementary file 2 [file oncotarget-08-83343-s002.pdf]

Reverse phase protein array (RPPA) combined with computational analysis to unravel relevant prognostic factors in non- small cell lung cancer (NSCLC): a pilot study

Supplementary Data

The Python code is available to the public repository:
https://github.com/fortunatobianconi/Oncotarget2017

Before you use follow this steps that are reported in README.md

###################################

1) Files description

###################################

This is the main code folder. It contains the following file:

1. case_report_model.py: ODEs implementation of the model
2. parameters.py: parameter values of the model
3. robustness_measure.py: script for parameter perturbation and parallel implementation of model_sim.py
4. model_sim.py: integration of the model and computation of the evaluation functions
5. memmap.py: optimization function for sharing data between processes (workers)
1. 6. base_hist_evalfunc.py: script for performing the intersection between evaluation functions tails pdf, for estimating conditional densities of parameters and for MIRI calculation
6. upper_lower_set.py: selection of the realizations of the parameter vector for which evaluation functions have higher or lower values
7. intersection.py: intersection between the evaluation functions tails pdf

###################################

2) Prerequisites

###################################

Before trying to use this code you must

first install the following packages:

numpy

pyDOE

joblib

pickle

scipy

tempfile

matplotlib

sklearn

collections

##################################

3) Usage

##################################

1. Run robustness_measure.py, setting the following parameters:

- LBpi and UBpi: lower and upper bound of the perturbed parameter space
- Nr and NSample: number of realizations and number of parameter vector samples for each realization
- ProteinNumber: chosen nodes for model calibration and/or validation
- fixed_p, fixed_Xt: indexes of parameters (kinetic parameters and total proteins) that do not have to be perturbed
- unfixed_x0: indexes of initial conditions to perturb
- input parameters of function Parallel, according to the performance of your device

This script calls the following functions: memmap and model_sim. Model_sim, in turn, calls parameters and case_report_model.

This script creates a pickle file containing all the evaluation functions measured.

2. Run base_hist_evalfunc.py, setting the following parameters:

- path of the filename containing the results
- name of the target region chosen for calibration and its opposite
- protein_name: names of the proteins selected for calibration and/or validation
- lowThr and highThr: thresholds for definition of lower and upper tails of the evaluation functions pdf

This script calls upper_lower_set and intersection.

It returns: MIRI for Nr realizations

CloudH_T: modes of the parameters pdf conditioned to the target region

CloudL_T: modes of the parameters pdf conditioned to the opposite region

##################################

4) OS and Python version

#################################

This code has been tested on Ubuntu 16.04 LTS (64bit) using Python 2.7.12.
