# Supplementary material for: Reverse phase protein array (RPPA) combined with computational analysis to unravel relevant prognostic factors in non- small cell lung cancer (NSCLC): a pilot study
Source: Oncotarget. 2017 Jun 14;8(47):83343–53. doi: 10.18632/oncotarget.18480 (PMC5669974; doi:10.18632/oncotarget.18480)
Supplement: Supplementary file 3 [file oncotarget-08-83343-s003.docx]

**Supplementary Table 1: list of the KEGG Pathways used to extend the model**

| **Protein** | **KEGG Pathway** |
| --- | --- |
| ERBB4 | ERBB Signaling pathway |
| cKIT | Pathways in cancer |
| CASP | Pathways in cancer |
| BAD | Pathways in cancer |
| LKB1 | AMPK Signaling pathway |
| AMPK | AMPK Signaling pathway |
| P70S6K | AMPK Signaling pathway |

**Supplementary Table 2: list of the 10 proteins selected in the RPPA dataset with low ratio (s-OS vs l-OS).**

| **Proteins having LOW ratio in the RPPA dataset** | **Ratio** |
| --- | --- |
| Smad2_S245_250_255 | 0.9556 |
| Cox2 | 0.148377 |
| NQO1 | 0.169314 |
| Bak | 0.198295 |
| bRaf_S445 | 0.239788 |
| Tubulin_alpha_acetylated | 0.297899 |
| p70S6Kinase_S371 | 0.343008 |
| Erk_T202_Y204 | 0.373439 |
| EGFR | 0.382893 |
| Stat3_S727 | 0.398519 |

**Supplementary Table 3: List of the 10 proteins selected in the RPPA dataset with high ratio (s-OS vs l-OS).**

| **Proteins having HIGH ratio in the RPPA dataset** | **Ratio** |
| --- | --- |
| EGFR_Y1148 | 1.698932 |
| LKB1_S334 | 1.768267 |
| Caspase3_Cleaved | 1.778907 |
| Caspase9_Cleaved | 1.78247 |
| ErbB4_Her4 | 1.879488 |
| cKit_Y719 | 2.042143 |
| AMPKbeta1_S108 | 2.840556 |
| cKit_Y703 | 3.553737 |
| Heme_Oxygenase1 | 1.12E+08 |
| BAD_S155 | 3.52E+08 |

**Supplementary Table 4: List of the proteins selected in the RPPA dataset. The ﬁrst column depicts proteins that were already in the original model, the second column shows those insertedusingKEGGandtheliteraturewhileinthethirdcolumntherearethoseexcluded from the model because not related with it.**

| **Proteins selected already in [1]** | **Proteins included from the literature and KEGG** | **Proteins not included in the model** |
| --- | --- | --- |
| EGFR | ERBB4 | SMAD2 |
| RAF | cKIT | COX2 |
| ERK | CASP | NQO1 |
|  | BAD | BAK |
|  | LKB1 | Tubulin-alpha-acetylated |
|  | AMPK | STAT3 |
|  | p70S6k | Heme-Oxygenase1 |
|  | mTOR |  |

**Supplementary Table 5: Initial concentrations of model species and total proteins amount. In the table,** $\boldsymbol{x}_{\boldsymbol{i}}\boldsymbol{i=1, \ldots,19}$ **represent state variables of the model while** $\boldsymbol{u}_{\boldsymbol{i}}$ **i = 1, ..., 3 are the input signals.**

| Variable | Name | Initial Value | Total Concentration |
| --- | --- | --- | --- |
| $x_{1}$ | EGFR* | 8000 | - |
| $x_{2}$ | IGF1R* | 8000 | - |
| $x_{3}$ | ERBB4* | 8000 | - |
| $x_{4}$ | cKIT* | 8000 | - |
| $x_{5}$ | SOS | 0 | - |
| $x_{6}$ | DSOS | 120000 | - |
| $x_{7}$ | Ras* | 0 | 120000 |
| $x_{8}$ | Raf* | 0 | 120000 |
| $x_{9}$ | Mek* | 0 | 600000 |
| $x_{10}$ | Erk* | 0 | 600000 |
| $x_{11}$ | p90* | 0 | 120000 |
| $x_{12}$ | PIK3* | 0 | 120000 |
| $x_{13}$ | Akt* | 0 | 120000 |
| $x_{14}$ | LKB1* | 0 | 360000 |
| $x_{15}$ | AMPK* | 0 | 360000 |
| $x_{16}$ | mTOR* | 0 | 360000 |
| $x_{17}$ | p70S6K* | 0 | 360000 |
| $x_{18}$ | Bad* | 120000 | - |
| $x_{19}$ | CASP* | 120000 | - |
| $u_{1}$ | RafPP | 120000 | - |
| $u_{2}$ | PP2A | 120000 | - |
| $u_{3}$ | RasGapActive | 120000 | - |

**Supplementary Table 6: List of kinetic parameters of the extended model.**

| **Parameter** | **Name** | **Value** |
| --- | --- | --- |
| p1 | γEGFR | 0.02 |
| p2 | γIGF1R | 0.02 |
| p3 | γERBB4 | 0.02 |
| p4 | γcKIT | 0.02 |
| p5 | γPIK3 | 0.0050000 |
| p6 | kp90Rsk:Erk | 0.0213697 |
| p7 | Kmp90Rsk:Erk | 763523.0000000 |
| p8 | kSOS:EGFR | 694.7300000 |
| p9 | KMSOS:EGFR | 6086070.0000000 |
| p10 | kSOS:ERBB4 | 7.0000000 |
| p11 | KMSOS:ERBB4 | 400000.0000000 |
| p12 | kSOS:cKIT | 7.0000000 |
| p13 | KMSOS:cKIT | 400000.0000000 |
| p14 | kRas:SOS | 32.3440000 |
| p15 | KMRas:SOS | 35954.3000000 |
| p16 | kErk:MEK | 9.8536700 |
| p17 | KMErk:MEK | 1007340.0000000 |
| p18 | kDSOS:p90Rsk | 161197.0000000 |
| p19 | KMDSOS:p90Rsk | 896896.0000000 |
| p20 | kSOS:IGF1R | 500.0000000 |
| p21 | KMSOS:IGF1R | 100000.0000000 |
| p22 | kPIK3:IGF1R | 10.6737000 |
| p23 | KMPIK3:IGF1R | 184912.0000000 |
| p24 | kPIK3:EGFR | 10.6737000 |
| p25 | KMPIK3:EGFR | 184912.0000000 |
| p26 | kAkt:PIK3 | 0.0566279 |
| p27 | KMAkt:PIK3 | 653951.0000000 |
| p28 | γAkt | 0.0050000 |
| p29 | kErk:PP2A | 8.8912000 |
| p30 | KMErk:PP2A | 3496490.0000000 |
| p31 | kERBB4:PIK3 | 7.0000000 |
| p32 | KMERBB4:PIK3 | 400000.0000000 |
| p33 | kcKIT:PIK3 | 7.0000000 |
| p34 | KmcKIT:PIK3 | 400000.0000000 |
| p35 | kPIK3:Ras | 0.0771067 |
| p36 | KMPIK3:Ras | 272056.0000000 |
| p37 | kRaf:Ras | 0.8840960 |
| p38 | KMRaf:Ras | 62464.6000000 |
| p39 | kMEK:Raf | 185.7590000 |
| p40 | KMMEK:Raf | 4768350.0000000 |
| p41 | kRaf:Akt | 15.1212000 |
| p42 | KMRaf:Akt | 119355.0000000 |
| p43 | kRas:RasGap | 1509.3600000 |
| p44 | KMRas:RasGap | 1432410.0000000 |
| p45 | kMEK:PP2A | 2.8324300 |
| p46 | KMMEK:PP2A | 518753.0000000 |
| p47 | kRaf:RafPP | 0.1263290 |
| p48 | KMRaf:RafPP | 1061.7100000 |
| p49 | γp90Rsk | 0.0050000 |
| p50 | kErk:LKB1 | 5.0000000 |
| p51 | KMErk:LKB1 | 1500000.0000000 |
| p52 | kLKB1:AMPK | 5.0000000 |
| p53 | KMLKB1:AMPK | 1500000.0000000 |
| p54 | kAMPK:mTOR | 7.0000000 |
| p55 | KMAMPK:mTOR | 400000.0000000 |
| p56 | kAkt:mTOR | 7.0000000 |
| p57 | KMAkt:mTOR | 400000.0000000 |
| p58 | kmTOR:p70S6K | 7.0000000 |
| p59 | KmmTOR:p70S6K | 400000.0000000 |
| p60 | kAkt:AMPK | 7.0000000 |
| p61 | KMAkt:AMPK | 400000.0000000 |
| p62 | kAkt:Bad | 7.0000000 |
| p63 | KMAkt:Bad | 400000.0000000 |
| p64 | kAkt:CASP | 7.0000000 |
| p65 | KMAkt:CASP | 400000.0000000 |

**Supplementary Table 7: List of the 10 calibrated parameters.** The ﬁrst column show names of the parameters ﬁxed using the calibration procedure, while the second and the third column represent the corresponding values in the s-OS and l-OS patient, respectively.

| **Parameter Name** | **Value s-OS** | **Value l-OS** |
| --- | --- | --- |
| γcKIT | 0.0270 | 0.1698 |
| kAkt:PIK3 | 0.0695 | 0.3532 |
| kRaf:Ras | 1.7494 | 6.6215 |
| kRas:RasGap | 11304.0000 | 1852.3000 |
| kAkt:Bad | 10.3443 | 49.7965 |
| PIK3tot | 132240.0000 | 943820.0000 |
| p70S6ktot | 261460.0000 | 3011800.0000 |
| RasGap* | 958860.0000 | 132240.0000 |
| cKIT*_0 | 68934.0000 | 5810.1000 |
| BAD*_0 | 1049000.0000 | 87158.0000 |

**Supplementary Table 8: Values of Closeness Centrality and Eccentricity computed by CentiScaPe, a plug-in of Cytoscape, for candidates proteins of the validation set.**

| **Protein** | **Closeness Centrality (CC)** | **Eccentricity (E)** | **CC+E** |
| --- | --- | --- | --- |
| ERK | 0.018 | 0.25 | 0.268 |
| AMPK | 0.018 | 0.2 | 0.218 |
| mTOR | 0.017 | 0.2 | 0.217 |
| LKB1 | 0.016 | 0.2 | 0.216 |
| MEK | 0.016 | 0.2 | 0.216 |

**Supplementary Table 9: Names of the measured proteins not used to calibrate the model. Only the last three proteins were selected for the validation process.**

|  | **Measured proteins not used for calibration** |
| --- | --- |
| Proteins excluded for model validation | EGFR  IGF1R  ERBB4  CASP  MEK  LKB1 |
| Validation set | ERK  AMPK  mTOR |
